# Hyperspectral Fingerprints of Abdominal and Pelvic Organs

**DOI:** 10.3390/jimaging12060262

**Published:** 2026-06-15

**Authors:** Laurie S. van de Weerd, Nick J. van de Berg, L. Lucia Rijstenberg, Ralf L. O. van de Laar, Heleen J. van Beekhuizen

**Affiliations:** 1 Department of Gynaecological Oncology, Erasmus Medical Center Cancer Institute, University Medical Center Rotterdam, 3015 GD Rotterdam, The Netherlands; 2 Department of BioMechanical Engineering, Delft University of Technology, 2628 CD Delft, The Netherlands; 3 Department of Pathology, Erasmus University Medical Center Rotterdam, 3015 GD Rotterdam, The Netherlands

**Keywords:** hyperspectral imaging, organ recognition, deep learning

## Abstract

Ovarian cancer (OC) is typically treated with cytoreductive surgery (CRS). Hyperspectral imaging (HSI) is an emerging non-invasive, label-free technique that enables whole-area scanning, making it a promising tool for real-time tumour recognition. However, developing tumour recognition algorithms requires a foundational understanding of spectral variability in normal tissues. This study focusses on the in vivo spectral profiles of key abdominal and pelvic organs encountered during CRS, including the uterus, ovaries, intestines, mesentery, omentum, peritoneum, and fallopian tubes, and evaluates the potential for organ recognition using HSI data. Intraoperative HSI data were from healthy patients. Two machine learning models, a support vector machine (SVM) and a 3D convolutional neural network (3DCNN), were trained to classify the organs based on their spectral signatures. In total, 15 patients were included in the dataset. The 3DCNN slightly outperformed the SVM in terms of the average accuracy (0.889 vs. 0.878), sensitivity (0.648 vs. 0.604), specificity (0.936 vs. 0.930), and Dice Similarity Coefficient (0.595 vs. 0.569). This study demonstrates the feasibility of using HSI for organ differentiation in the clinical setting, although in some cases separability remains a challenge, especially when organs have similar spectra. This is a critical step towards a generalizable in vivo abdominal tumour recognition algorithm, by carefully investigating spectral fingerprints of abdominal tissues.

## 1. Introduction

Ovarian cancer (OC) is the eighth-most occurring cancer among women [1]. Of OC cases, 90% are epithelial ovarian cancers (EOC), with the remaining 10% being of non-epithelial origin [2]. Approximately 80% of EOCs are diagnosed at an advanced stage, as the patients tend to present non-specific symptoms that do not form a clear or easily recognizable pattern [3]. Due to this late presentation, OC is sometimes called the silent lady killer [4]. Standard treatment typically consists of cytoreductive surgery (CRS) and chemotherapy, followed by maintenance therapy in selected patients. In about 85% of the cases, patients receive three cycles of neoadjuvant chemotherapy (NACT) followed by a CT-scan evaluation, followed by CRS in patients with stable disease or remission, followed again by three cycles of chemotherapy. The primary goal of CRS is complete resection of all macroscopic disease. Any tissue that is suspected of containing tumour cells is resected. Extensive surgery may involve dissection of enlarged lymph nodes, removal of tumour lesions at vulnerable locations, or multiple bowel anastomoses. As CRS is highly invasive, morbidity and mortality rates are significant. Improving surgical accuracy and reducing operation time and blood loss can mitigate some of these risks [5].

For patients receiving NACT before CRS, fibrosis of previous malign tissue may be present. Since fibrotic tissue can resemble tumour tissue, unnecessary fibrotic tissue and/or lymph node resections can significantly prolong CRS without benefit. By selectively resecting vital tumour lesions only, operation time and blood loss may be decreased, and organ damage may be reduced. An intraoperative tumour recognition method could help avoid unnecessary resections while still achieving complete CRS.

Hyperspectral imaging (HSI) is an emerging technique in healthcare, providing a non-invasive, radiation-free, label-free method for scanning whole tissue areas at once. Materials reflect, absorb, and emit light in specific patterns at specific wavelengths due to their unique chemical composition and physical structure. An HSI camera captures a sequence of images for different wavelengths and measures the intensity of reflected light across these wavelengths, producing a reflectance spectrum for each pixel. These spectra can serve as unique fingerprints for different tissue types [6,7]. In combination with machine learning (ML) or deep learning (DL) algorithms, HSI could become a valuable tool for tumour recognition.

Numerous studies have looked into the possible use of HSI as a tumour recognition tool, investigating various cancers, including breast, head and neck, and gastric cancers [8,9,10]. Typically, these studies typically utilized ex vivo tissue samples [8]. Perez et al. demonstrated that HSI could detect EOC ex vivo with a sensitivity of 0.81 and specificity of 0.70 [11]. A critical next step is to determine whether HSI can be used intraoperatively to differentiate between tissue types. Collins et al. investigated the in vivo detection of colon and esophagogastric cancer with HSI. A ROC-AUC value of 0.93 was found by combining HSI data with a 3D convolutional neural network (3DCNN) [12].

Developing tumour recognition algorithms requires a foundational understanding of the spectral variability present in normal tissues. Although HSI is often described as “fingerprint-like” data, it is unknown how distinct these fingerprints truly are among adjacent organs, many of which share similar composition or embryologic origin. Most previous work have focused on single tissues or preselected ex vivo samples, meaning that inter-organ separability in intraoperative conditions remains largely untested.

Our study addresses this gap by characterizing the in vivo spectral profiles of key organs encountered during CRS, including the uterus, ovaries, intestines, mesentery, omentum, peritoneum, and fallopian tubes. As a tissue-characterization study, the focus is not on applied tumour-detection analysis, but on evaluating the ability to identify organs from HSI data alone, using only their spectral and local textural signatures, without relying on anatomical or topological context. This establishes whether organs can be pooled under a single “healthy tissue” category for future algorithm development, or whether training must include balanced representation from each anatomical site to prevent bias and loss of generalizability. This choice directly addresses the trade-off between model granularity and generalizability: while more classes increase detail, they also complicate the learning task and increase the required dataset size. In this sense, by characterizing the spectral variance of healthy tissues, we define the necessary baseline against which future models must distinguish pathology.

## 2. Materials and Methods

**Figure 1:**
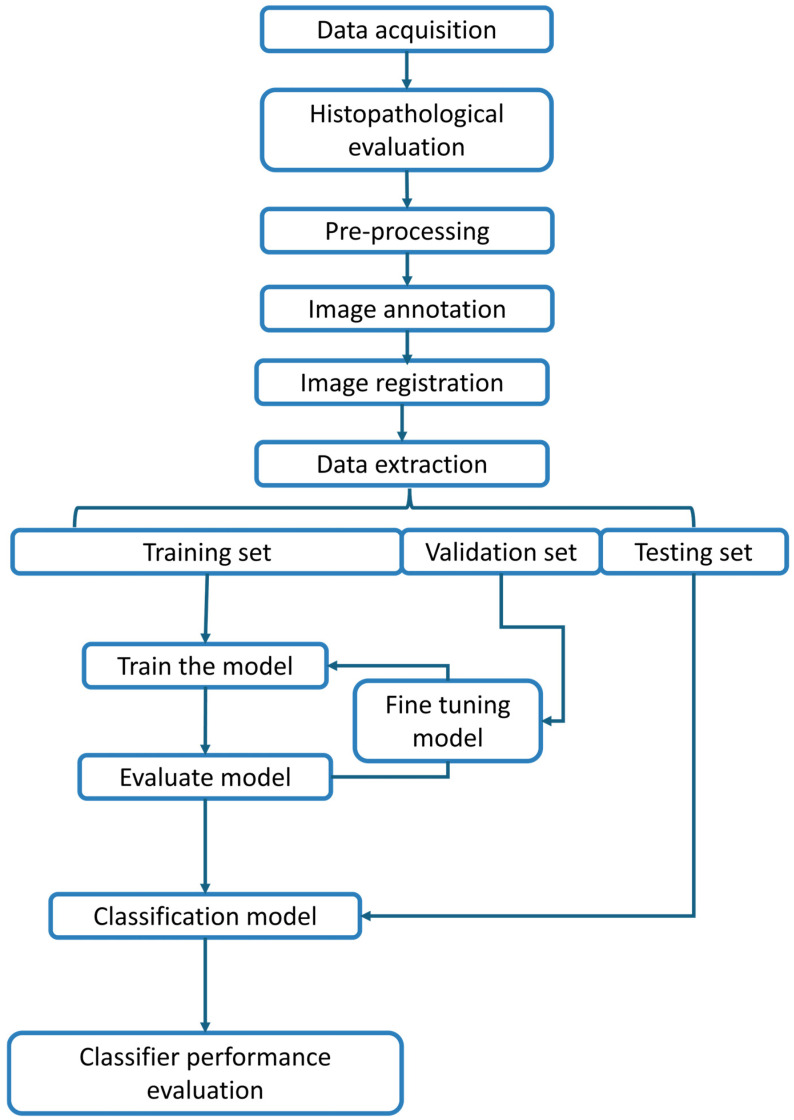
Overview of the methodology. Classification model refers to the SVM and the 3DCNN.

An overview of the study methodology can be found in Figure 1.

### 2.1. Participants

Participants were recruited at the Erasmus University Medical Center in Rotterdam, the Netherlands. Patients aged 18 years or above with low stage cervical or endometrial cancer were scheduled for either a Wertheim procedure (radical hysterectomy) or a laparotomy. Only patients in whom no intra-abdominal tumour was present during surgery were included. Informed consent was obtained from all participants. The study was carried out according to the standards outlined in the Declaration of Helsinki. All procedures involving patients were approved by the Medical Ethical Committee of the Erasmus Medical Center, Rotterdam, in the Netherlands (trial protocol MEC-2021-0902).

### 2.2. Instrumentation

The HSI system that was used was the TIVITA 2.0 camera (Diaspective Vision GmbH, Am Salzhaff, Germany). The camera operates with a pushbroom scanning system and has a spatial resolution of 640 × 480 pixels. It provides a spectral resolution of 5 nm, covering a spectral range of 500–1000 nm, and uses LED lights as the illumination source. The acquisition time is approximately 6.4 s. The working distance is 50 cm, and the field of view (FOV) is 8.0 × 5.9 cm, with an approximate spatial resolution of 0.12 mm/pixel. The system captures the reflectance spectra per pixel on a metal oxide semiconductor (CMOS) image sensor, simultaneously recording HSI data and an RGB image on two different sensors.

### 2.3. Data Acquisition

The gynecological oncologist positioned the camera at a working distance of 50 cm, ensuring that the organs were in focus and visible within the FOV. The imaged organs included the uterus, ovaries, intestines, mesentery, omentum, peritoneum, and fallopian tubes.

During the surgical procedure, tissue was resected and/or lymph nodes were removed as part of routine care. After the surgery, these specimens were sent to the pathology department and processed in the standard manner for diagnostic histopathology. The tissue was stained with hematoxylin and eosin (H&E) and digitized. The pathologist then reviewed the digitized images and marked the tissue and/or nodes as either tumour-positive or tumour-negative. Patients were included only when all resected tissue samples or nodes were tumour-negative and no cancer was detected intra-abdominally.

### 2.4. Data Pre-Processing

All HSI data were pre-processed by performing image calibration, glare pixel removal, and noise filtering, respectively.

#### 2.4.1. Image Calibration

Image calibration was conducted to correct for signal variations caused by non-uniform illumination and pattern noise [13]. The images were calibrated using a white and a black reference image. The white reference image was made using a Spectralon 99% sample (SRS-99-010, Labsphere Inc., Northern Sutton, NH, USA), while the black reference image was captured by blocking all the incident light with a thick piece of black paper. The white sample was segmented in the acquired images, and the average intensity of the selected region of interest (ROI) per wavelength was calculated. The calibrated image was computed by:

(1) $$I_{cal}\left(x,y,\lambda \right)=\frac{I_{raw}\left(x,y,\lambda \right)-I_{dark}(x,y,\lambda )}{I_{white}\left(x,y,\lambda \right)-I_{dark}(x,y,\lambda )}$$

In this equation, $I_{cal}\left(x,y,\lambda \right)$ represents the calibrated image; $I_{raw}\left(x,y,\lambda \right)$ the raw image; $I_{dark}(x,y,\lambda )$ the dark reference image; and $I_{white}\left(x,y,\lambda \right)$ the white reference image. Here, (x,y) denotes the pixel coordinates and λ represents the wavelength band.

#### 2.4.2. Data Cleaning and Signal Conditioning

Glare pixels were eliminated using intensity thresholding. The mean intensity per wavelength was calculated along with the standard deviation. Pixels with values exceeding the mean intensity plus five times the standard deviation were removed from the ROI. The spectra were filtered by use of a 2nd order low-pass Butterworth filter with a cutoff frequency of 0.3. This filter was used to remove sharp peaks caused by noise in the spectra; however, the filter parameters were carefully selected to suppress this non-biological noise while preserving the underlying spectral pattern. Additionally, normalization was used to scale the spectra.

### 2.5. Classification

#### 2.5.1. Image Annotation

After image acquisition, the RGB images were annotated by experienced gynecological oncologists. The annotations included the uterus, ovaries, intestines, mesentery, omentum, peritoneum, and fallopian tubes. Annotations were only made when the organ type was clearly identifiable in the image. Margins were taken at the boundaries between different organs to ensure a clean dataset.

#### 2.5.2. Image Registration

After this, image registration was performed to overlay the RGB with the HSI data. Image registration was done manually with Photoshop. One of the images from the hypercube was selected in MATLAB R2024b (MathWorks, Natick, MA, USA) and imported into Adobe Photoshop 2021 (version 22.0; Adobe Inc., San Jose, CA, USA), along with the annotated RGB image. Two reference points were chosen in the HSI image, and the RGB image was aligned to the HSI image through scaling, rotation, and translation. Visual inspection was performed to ensure that the selected regions overlapped well between the registered images. After aligning the RGB image and the HSI data, different organs were selected via Hue Saturation Value (HSV) thresholding. This HSV thresholding was used to create a binary mask that could be multiplied with the HSI data. This process allowed for the selection and labelling of different tissue types. Features were then extracted from the pixels identified through the binary mask.

#### 2.5.3. Classification Models

Two classification models were applied to the HSI data, including a support vector machine (SVM) and a three-dimensional convolutional neural network (3DCNN).

##### Support Vector Machine (SVM)

SVMs are relatively straightforward to train and perform well when working with datasets that are limited in size but high-dimensional [14]. An SVM aims to identify the optimal decision boundary or hyperplane that maximizes the margin between different classes. For problems that are not linearly separable, kernel functions are used to project the data into a higher-dimensional feature space where linear separation becomes possible [15].

In this study, a one-vs-one strategy is applied to solve the multi-class classification problem. This approach decomposes the multi-class problem into multiple binary classification tasks, training a separate SVM for each pair of classes. A final prediction is determined by combining the outcomes of these binary classifiers. A grid search was performed to determine the optimal kernel type and the value of the regularization parameter. Additionally, class weights were used for handling the class imbalance.

##### Convolutional Neural Network (CNN)

CNNs are multi-layer neural networks that can identify, recognize, and classify objects, as well as detect and segment objects in images [16]. Generally, a CNN is composed of a collection of neurons organized in layers, each with learnable weights and biases. To use a CNN for image recognition, it must be trained on a dataset of labelled images containing the objects of interest. The CNN learns to associate extracted features with correct labels through a process of backpropagation and optimization. Once trained, it can be used to make predictions based on new data [17].

A 3DCNN was adapted from a previously published model and modified to suit the characteristics of our multi-class tissue classification task [18]. Seven hidden layers were used, of which the first 6 layers were convolutional. Each convolutional block consisted of a convolutional layer followed by batch normalization and a ReLU activation function, enabling effective feature extraction and learning. Additionally, striding and pooling operations are used in certain layers to gradually decrease the spatial dimensions, while preserving key features. The last hidden layer was a fully connected layer that consisted of 7 neurons, as there were 7 tissue classes to predict. Lastly, a Softmax layer and classification layer with added class weights were implemented to account for the class imbalance. A complete overview of the used 3DCNN can be found in Table 1.

#### 2.5.4. Implementation, Dataset Split, and Training

The data was divided into training, validation, and test sets with k-fold cross-validation (k = 5). In each fold, 9 patients were used in the training set, 3 patients in the validation set, and 3 patients in the test set. This ensured that all tissue classes were present in training, validation, and test sets and that all patients were in the test set once.

Relative reflectance spectra of different organs were used as an input for the SVM; therefore, the feature space had 100 dimensions (one dimension per wavelength). For the 3DCNN, the input data consisted of small sub-volumes of size 5 × 5 × 100. These sub-volumes were generated using a sliding window technique with a step of one. At the edges of the ROI, padding was applied. The mirroring method was used, where the data at the edges was reflected to extend the input volume. This was done to ensure that every pixel in the ROIs could be used without losing information at the boundaries.

The 3DCNN was trained using the Adam optimizer with an initial learning rate of 0.001 and momentum of 0.9. A piecewise learning rate schedule was applied with a learning rate drop factor of 0.01 every 30 epochs. The model was trained using 5 epochs and a mini-batch size of 64. The training data were shuffled at every epoch to improve generalization. Gradient clipping based on the L2 norm was applied. Training was initialized with a fixed random seed to ensure reproducibility.

#### 2.5.5. Classifier Performance Evaluation

The performance of the classifier was evaluated by use of evaluation metrics, including the confusion matrices, accuracy, sensitivity, specificity, the DICE similarity score, and the area under the receiver operator curve (AUC-ROC). These metrics were calculated for each test set. The overall scores were determined by averaging metrics across the five folds.

## 3. Results

### 3.1. Participants

Between March 2023 and April 2025, a total of 15 patients were included in the study. The patient characteristics can be found in Table 2. The included patients were treated for five different types of diseases, including a cystic enlarged ovary (*n* = 1), serous adenofibroma (*n* = 1), cervical squamous cell carcinoma (*n* = 10), serous endometrial carcinoma (*n* = 1), and cervical adenocarcinoma (*n* = 2).

### 3.2. Spectral Data

**Figure 2:**
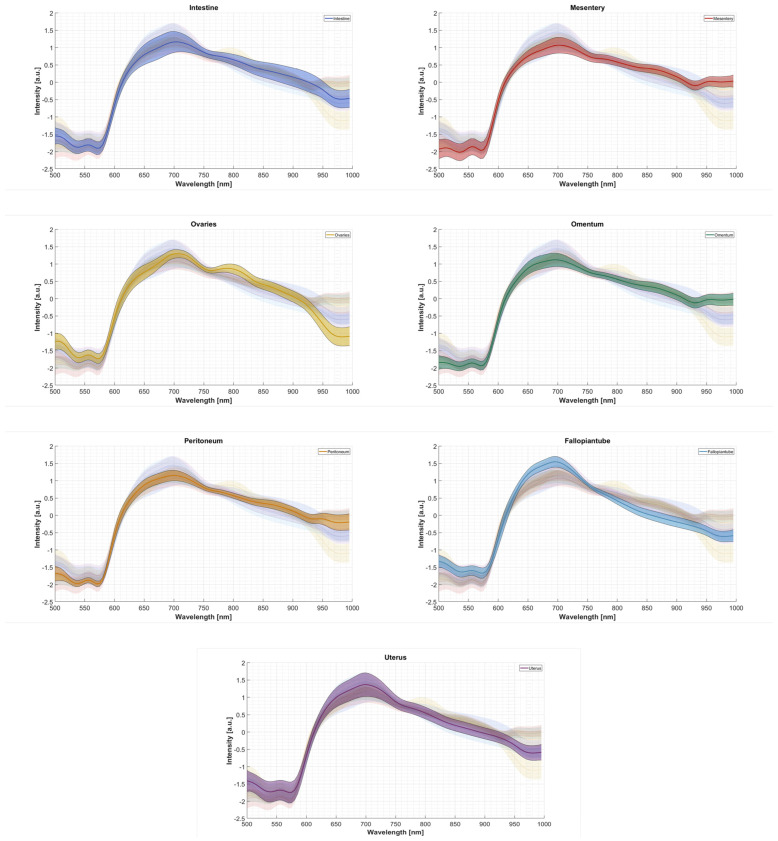
Spectral curves of the different tissue classes. The intensity is plotted as a function of the wavelength for each class. The mean spectral curve is shown as a thick solid line in the middle of the shaded area; the shaded area covers the mean +/− 1 SD.

Spectral curves of the different organs are visualized in Figure 2. In this figure, the mean intensity per organ, together with the standard deviation, can be seen. As a reference, spectral curves of the other organs are shown in the background. There is a considerable amount of spectral overlap for the different organs.

### 3.3. Classifier Performance Evaluation

**Figure 3:**
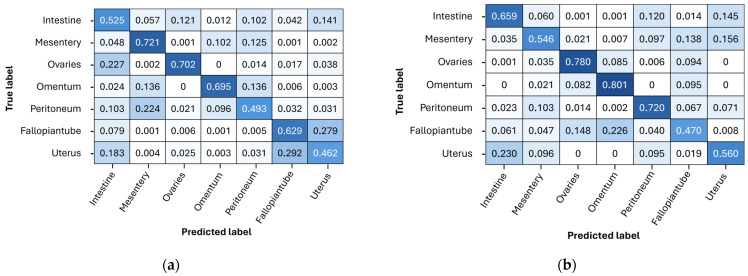
Confusion matrices of the (**a**) SVM and (**b**) 3DCNN model. The matrices are normalized, so each row adds up to a value of one. The rows correspond to the true labels, and the columns correspond to the predicted tissue types by the models. The diagonal values represent the proportion of pixels that are correctly predicted.

Figure 3 presents the confusion matrices for the two models. Both matrices are normalized, meaning that the values in each row add up to 1. A confusion matrix provides a summary of a classification model’s performance. In this seven-way organ classification problem, the confusion matrix for the SVM model (Figure 3a) shows the highest sensitivity of 72.1% for the mesentery and the lowest sensitivity of 46.2% for the uterus. The uterus and fallopian tube classes are often misclassified (29.2% and 27.9% of data points, respectively). For the 3DCNN (Figure 3b), the highest sensitivity of 80.1% was achieved for the omentum, and the lowest sensitivity of 47.0% for the fallopian tube.

Table 3 presents the mean accuracy, sensitivity, specificity and DICE score per organ for the two models, along with their standard deviations. For the SVM model, the average accuracy across all organs was 0.878; the highest accuracy was achieved for the ovaries (0.939), while the lowest accuracy was for both the intestine (0.829) and the uterus (0.829). The average sensitivity across all organs was 0.604, where the highest sensitivity was observed for the mesentery (0.721), while the lowest sensitivity was for the uterus (0.462). Regarding specificity, the average value across all organs equals 0.930, where the highest value was obtained for the ovaries (0.970), and the lowest for the intestine (0.888). The average DICE score across all organs equals 0.569, with the highest DICE score observed for the omentum (0.706) and the lowest for the peritoneum (0.430).

For the 3DCNN model, the average accuracy across all organs was 0.889, with the highest accuracy achieved for the omentum (0.931), and the lowest accuracy for the mesentery (0.862). The average sensitivity was equal to 0.648, where the highest sensitivity was observed for the omentum (0.801), while the lowest sensitivity was for the fallopian tube (0.470). The average specificity across all organs was equal to 0.936, with the highest specificity observed for the ovaries (0.950) and the lowest specificity for the mesentery (0.925). Lastly, the average DICE score was equal to 0.595, with the lowest DICE score observed for the fallopian tube (0.341) and the highest DICE score for the ovaries (0.758).

**Figure 4:**
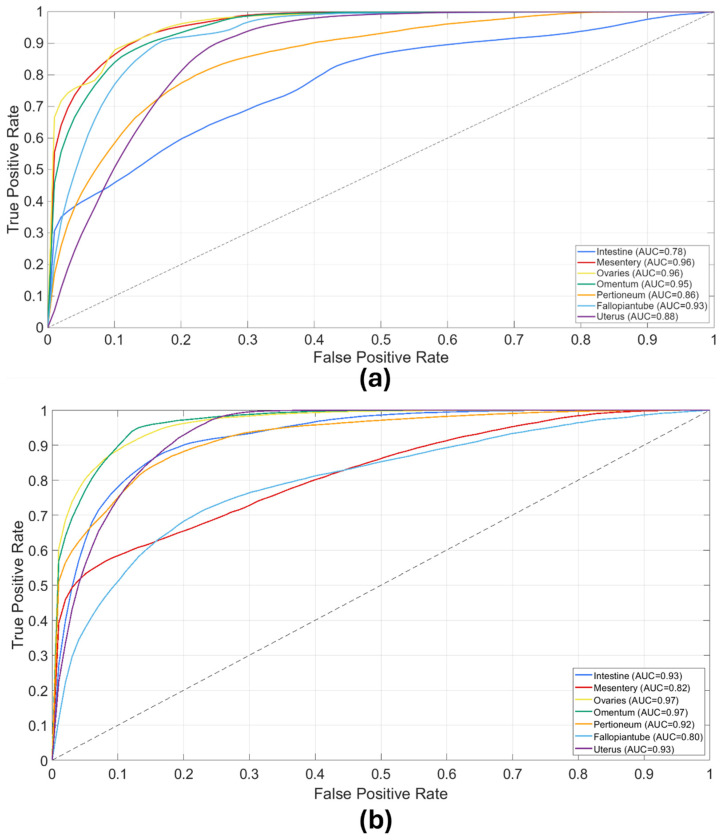
ROC curves for the (**a**) SVM model and the (**b**) 3DCNN model in classifying the seven different organs. The AUC values for each organ are indicated in the legend. The dashed diagonal line represents the performance of a random classifier (AUC = 0.5).

Furthermore, the ROC curves were plotted to assess the model’s performance in terms of false positive and true positive rates (see Figure 4). The area under the curve (AUC-ROC) provides a measure of classification performance, with values closer to 1 indicating better performance. For the SVM model, all the ROC-AUCs are above 0.93, except for the intestine (AUC = 0.78) and the uterus (AUC = 0.88), which indicates a lower recognition performance for these two organs.

For the 3DCNN, all the ROC-AUCs were above 0.92, except for the mesentery (AUC = 0.82) and the fallopian tube (AUC = 0.80).

## 4. Discussion

In this study, we demonstrate that seven different tissue types in the human abdominal region can be recognized in vivo by combining HSI with machine learning techniques. During CRS for ovarian cancer, disease spread often involves multiple abdominal and pelvic organs. In the development of robust HSI-based algorithms that detect pathological signatures, it is essential to first establish a baseline for healthy inter-organ spectral variability. Without such a baseline, tumour classifiers may overfit to surgical settings, confound organ type with pathology, and perform inconsistently across different patients and clinical centres.

The results highlight the considerable spectral variability between different tissues but also underscore the challenge of distinguishing adjacent organs with similar compositions. The 3DCNN demonstrated slightly higher average performance metrics compared to the SVM. However, these differences fall within the variability observed across cross-validation folds and should therefore not be interpreted as statistically significant. Given the limited sample size and inter-patient heterogeneity, the reported performance differences should be interpreted with caution. In addition, the ROC curves reflect variability introduced by the limited number of patients and uneven class distribution. As a result, the shape and stability of the ROC-AUC estimates vary across cross-validation folds, indicating uncertainty in the reported performance estimates rather than precise model discrimination ability.

One of the strengths of our study lies in the use of HSI in vivo clinical conditions in humans, which is still not the standard in research involving HSI. Data acquisition in a surgical environment comes with limitations, such as tissue accessibility, varying view angles depending on surgeon or camera positions, moist tissue surfaces causing specular reflection, and the lighting conditions typical of open abdominal surgeries. Additionally, the dynamic nature of in vivo imaging, including respiratory motion and surface reflections, can significantly impact data quality. The value of this study lies in evaluating HSI in the conditions in which it is ultimately intended to be used: during live surgery. Beyond tissue characterization, the broader aim is to investigate whether HSI can contribute to intraoperative guidance from this practical viewpoint, ultimately aiming to support intraoperative decision-making and improve surgical precision.

Although we observe differences between the spectral fingerprints of organs, some tissue types have more resemblance than others. These variations in spectral signatures are closely related to underlying physiological and biochemical tissue properties. Differences in blood perfusion influence absorption characteristics, particularly in the wavelength regions associated with hemoglobin (around 542 nm and 577 nm), while variations in lipid content primarily affect the near-infrared range. In addition, tissue structure and density also contribute to differences in spectral profiles [19]. These underlying factors help explain why some tissues are more difficult to distinguish than others. Statistically, two factors can complicate organ delineation: similarity in mean spectral curve and high spectral variability.

For example, the fallopian tubes and uterus have comparable spectral curves. This resemblance might be explained by their shared embryological origin, their anatomical continuity, and similar imaging characteristics [20]. From both a clinical and computational perspective, these tissues have a similar baseline. In ML terms, they could potentially be placed into a single class.

In turn, the intestines were characterized by considerable variations in spectral signatures. When visually inspecting the images, intestinal tissues could present observable differences in colour, (fecal) filling, surface geometry (folds, ridges, shadowed regions), and the presence of adipose tissue surrounding the intestine. These macroscopic differences were reflected in the spectral signatures, which showed great variability.

These observations were consistent with the classification results. The largest misclassifications occurred between the ovaries and the intestine, and between the fallopian tubes and the uterus.

For the intestine, misclassifications were distributed across the ovaries, peritoneum, and uterus. This dispersion may be attributed to the large variability observed in the intestinal spectral signatures. Additionally, substantial differences in intestinal tension were observed between patients, potentially caused by varying levels of intraluminal contents. Increased tissue stress may alter optical properties, like translucency, further contributing to spectral variability.

In addition to these challenging cases, certain tissue classes demonstrated consistently higher classification performance. The relatively high accuracy observed for the ovary and omentum classes may be explained by intrinsic tissue properties; the omentum, which is rich in adipose tissue, exhibits a characteristic spectral profile, particularly in the near-infrared range. Additionally, it often presents a distinct lobulated structure, which may further enhance spectral separability from surrounding tissues. Ovarian tissue, in contrast, often appears as a more visually compact and distinguishable structure compared to surrounding abdominal organs, characterized by a deeper red coloration, which may facilitate more robust spectral separation.

Similarities in healthy spectral baselines do not necessarily limit the development of tumour-detection algorithms. In fact, where healthy tissues exhibit overlapping spectra and can be grouped, this not only simplifies the learning problem but may also reduce confounding between organ identity and pathological signatures in downstream tumour-classification tasks. These findings indicate that while organ recognition based on spectral information is feasible, certain anatomically or compositionally related tissues remain difficult to distinguish. In selected cases, grouping related anatomical structures could improve robustness and clinical applicability.

The use of HSI in clinical settings is advancing, especially due to its non-invasive and label-free nature. Fischer et al. investigated in vivo organ recognition using HSI in a porcine model. Images were acquired in vivo and annotated retrospectively [21]. The same HSI camera was used as in our study, although illumination was provided by six halogen lamps instead of LED lights. A subset of 11 pigs underwent additional recordings to assess the impact of different sources of variability, including imaging angle, organ position, and repeated measurements. Linear mixed-effects modelling revealed that only the organ positioning and annotated regions explained a significant proportion of variance (13.8%), suggesting that HSI reflectance is primarily organ-specific rather than subject-dependent. Using these data, an ML-based organ recognition system achieved an average accuracy of 95.4% ± 3.6% [21]. In comparison, our study achieved an average accuracy of 87.8% and 88.9% for the SVM and the 3DCNN, respectively. This difference may be explained by several factors. First, the availability of repeated measurements per subject in the porcine study (196 photos/pig) allowed for improved signal stability and reduced intra-class variability. In the clinical setting of our study, acquiring multiple images per patient was often limited, as minimizing invasiveness and surgical delay was prioritized. Additionally, intraoperative data collection is inherently more challenging due to the presence of motion and specular artefacts; however, our study may better reflect-world clinical conditions, where variability is unavoidable and models must be robust to heterogeneous data acquisition settings.

Eggert et al. evaluated in vivo detection of head and neck tumours in humans using an endoscopic HSI system combined with deep learning [22]. In their cohort of 98 patients, a 3D DenseNet architecture achieved an average accuracy of 81%, with a sensitivity of 83% and specificity of 79%. A notable advantage of head and neck imaging compared to abdominal imaging is the reduced influence of respiratory motion, resulting in less distortion of the hyperspectral data cubes.

More recently, Bannone et al. evaluated HSI/based automatic abdominal tissue recognition in a prospective bi-centre study involving 169 patients undergoing open abdominal surgery [23]. Thirteen tissue types were classified, ranging from skin to veins. The best performance was achieved in within-center validation, with sensitivities of 100% for skin and 97% for liver. However, skin exhibits a markedly different spectral profile compared to intra-abdominal tissues, which limits direct comparability with our results. Similar to our findings, misclassifications were reported between omentum and mesentery (32%), whereas in our study this confusion was observed in approximately 10% of cases. These results suggest that while HSI has a high potential for abdominal tissue differentiation, challenges remain in distinguishing tissue types with similar spectral profiles.

A limitation of the study was that the small dataset was also substantially imbalanced, as not all seven tissue types could be imaged in every patient due to time constraints, anatomical absence, or unsuccessful measurements. In addition, variability across patients and limited cross-validation folds may have introduced instability in model evaluation. Some confidence intervals extended beyond the theoretical range of [0, 1], reflecting statistical uncertainty associated with small sample sizes and high variance across folds. These intervals should therefore be interpreted as indicators of uncertainty rather than precise performance bounds. Furthermore, while exact sample size requirements depend on the expected effect size and variance in performance metrics, a substantially larger cohort would be required to reliably compare classification results. Future work should therefore include formal power analyses and multicentre data collection to ensure adequately powered model comparisons. Overall, the reported results should not yet be generalized to clinical practice without validation in larger, and more balanced datasets.

## 5. Conclusions

To conclude, this work highlights the potential of HSI combined with machine learning for real-time, non-invasive abdominal tissue classification. While inter-organ separability remains a challenge, especially for organs with similar spectra, this study demonstrates the feasibility of using HSI for organ differentiation in the clinical setting. This study is exploratory in nature due to the limited sample size and should be interpreted accordingly. Given the limitations of current classification models, future work should focus on refining data acquisition techniques, addressing motion artefacts, and acquiring a larger, more balanced dataset. These improvements would enhance model generalizability and classification performance, paving the way for a more robust, clinically applicable tissue classification system. In addition, a better understanding of inter-organ spectral variability provides an important foundation for future intraoperative applications, where HSI may contribute to the identification of tumour lesions and assist in delineating resection margins during cytoreductive surgery for ovarian cancer. Subsequent tumour-focused validation studies are required before clinical translation can be achieved.

## Figures and Tables

**Table 1:** Detailed overview of the 3DCNN, including the layer types, kernel sizes, number of filters, and stride configurations.

Layer	Type	Kernel	Filters	Stride
Input	Image3D	5 × 5 × 100 × 1	-	-
Conv1	Conv3D	3 × 3 × 2	20	1 × 1 × 1
Conv2	Conv3D	2 × 2 × 3	20	2 × 1 × 1
Conv3	Conv3D	1 × 1 × 7	35	1 × 1 × 1
Conv4	Conv3D	1 × 1 × 9	35	2 × 1 × 1
Conv5	Conv3D	1 × 1 × 7	35	1 × 1 × 1
Conv6	Conv3D	2 × 2 × 3	35	2 × 1 × 1
FC	Fully Connected	-	numClasses	-
Softmax	Softmax	-	-	-
Output	Classification	-	7 classes	-

**Table 2:** Patient’s characteristics, surgical procedure, and retrieved data overview.

	Overall (*n* = 15)	Datapoints (% of Total)
***Patient’s characteristics***		
Age (years, median, range)	58 (35–80)	
BMI (kg/m^2^, median, range)	28.1 (18.9–38.1)	
***Surgical procedure (number of patients, %)***		
Wertheim (*n*, %)	13 (86.7%)	
Laparotomy (*n*, %)	2 (13.3%)	
***HSI data***		
Total number of extracted data points (*n*, %)	2,347,516 (100%)	
***Organs (number of patients for which organ was imaged %)***		
Intestine	10 (66.7%)	223,346 (9.5%)
Mesentery	11 (73.3%)	500,782 (21.3%)
Ovaries	10 (66.7%)	155,833 (6.6%)
Omentum	9 (60.0%)	986,339 (42.0%)
Peritoneum	8 (53.3%)	198,810 (8.5%)
Fallopian tube	9 (60.0%)	50,635 (2.2%)
Uterus	11 (73.3%)	231,772 (9.9%)

**Table 3:** Performance metrics of the SVM and 3DCNN models in terms of accuracy, sensitivity, specificity, and DICE score. Results are reported as the mean ± standard deviation, with 95% confidence intervals (CI) shown in brackets.

Accuracy	Intestine	Mesentery	Ovaries	Omentum	Peritoneum	Fallopian Tube	Uterus
**SVM**	0.829 ± 0.108 [0.695–0.963]	0.892 ± 0.035 [0.848–0.935]	0.939 ± 0.051 [0.875–1.003] *	0.921 ± 0.038 [0.873–0.968]	0.875 ± 0.041 [0.824–0.925]	0.871 ± 0.047 [0.813–0.929]	0.829 ± 0.032 [0.789–0.869]
**3DCNN**	0.890 ± 0.046 [0.832–0.947]	0.862 ± 0.061 [0.786–0.938]	0.918 ± 0.038 [0.871–0.966]	0.931 ± 0.038 [0.883–0.978]	0.896 ± 0.061 [0.820–0.971]	0.862 ± 0.041 [0.811–0.913]	0.865 ± 0.036 [0.820–0.909]
**Sensitivity**	**Intestine**	**Mesentery**	**Ovaries**	**Omentum**	**Peritoneum**	**Fallopian Tube**	**Uterus**
**SVM**	0.525 ± 0.247 [0.218–0.833]	0.721 ± 0.167 [0.514–0.927]	0.702 ± 0.375 [0.237–1.167] *	0.695 ± 0.194 [0.453–0.936]	0.493 ± 0.284 [0.140–0.846]	0.629 ± 0.220 [0.357–0.902]	0.463 ± 0.150 [0.277–0.649]
**3DCNN**	0.659 ± 0.179 [0.437–0.881]	0.546 ± 0.286 [0.190–0.901]	0.780 ± 0.155 [0.588–0.972]	0.801 ± 0.249 [0.492–1.110] *	0.720 ± 0.161 [0.521–0.920]	0.470 ± 0.394 [−0.020–0.960] *	0.560 ± 0.279 [0.214–0.907]
**Specificity**	**Intestine**	**Mesentery**	**Ovaries**	**Omentum**	**Peritoneum**	**Fallopian Tube**	**Uterus**
**SVM**	0.888 ± 0.112 [0.748–1.028] *	0.933 ± 0.066 [0.851–1.014] *	0.970 ± 0.025 [0.939–1.001] *	0.961 ± 0.025 [0.929–0.992]	0.929 ± 0.049 [0.868–0.990]	0.920 ± 0.052 [0.856–0.984]	0.910 ± 0.056 [0.841–0.979]
**3DCNN**	0.934 ± 0.043 [0.881–0.988]	0.925 ± 0.030 [0.888–0.962]	0.950 ± 0.041 [0.899–1.002] *	0.949 ± 0.043 [0.896–1.001] *	0.934 ± 0.063 [0.855–1.013] *	0.927 ± 0.065 [0.846–1.007] *	0.930 ± 0.068 [0.845–1.015] *
**DICE**	**Intestine**	**Mesentery**	**Ovaries**	**Omentum**	**Peritoneum**	**Fallopian Tube**	**Uterus**
**SVM**	0.491 ± 0.231 [0.204–0.778]	0.678 ± 0.086 [0.572–0.784]	0.680 ± 0.366 [0.225–1.134] *	0.706 ± 0.145 [0.525–0.886]	0.430 ± 0.201 [0.181–0.680]	0.536 ± 0.047 [0.477–0.595]	0.465 ± 0.082 [0.364–0.567]
**3DCNN**	0.590 ± 0.084 [0.486–0.695]	0.515 ± 0.217 [0.245–0.785]	0.758 ± 0.059 [0.685–0.831]	0.731 ± 0.142 [0.555–0.907]	0.678 ± 0.124 [0.525–0.832]	0.341 ± 0.238 [0.046–0.635]	0.551 ± 0.137 [0.381–0.722]

* *CI exceeded theoretical bounds and reflects estimation uncertainty from small sample size*.

## Data Availability

The original contributions presented in this study are included in the article. Further inquiries can be directed to the corresponding author.

## Abbreviations

The following abbreviations are used in this manuscript:

OC	Ovarian Cancer
EOC	Epithelial Ovarian Cancer
CRS	Cytoreductive Surgery
NACT	Neoadjuvant Chemotherapy
HSI	Hyperspectral Imaging
ML	Machine Learning
DL	Deep Learning
3DCNN	3D Convolutional Neural Network
FOV	Field of View
CMOS	Metal Oxide Semiconductor
H&E	Hematoxylin and Eosin
ROI	Region of Interest
SVM	Support Vector Machine
AUC-ROC	Area Under the Receiver Operator Curve

## References

[1] Sung H., Ferlay J., Siegel R.L., Laversanne M., Soerjomataram I., Jemal A., Bray F. (2021). Global Cancer Statistics 2020: GLOBOCAN Estimates of Incidence and Mortality Worldwide for 36 Cancers in 185 Countries. CA Cancer J. Clin..

[2] Momenimovahed Z., Tiznobaik A., Taheri S., Salehiniya H. (2019). Ovarian cancer in the world: Epidemiology and risk factors. Int. J. Women’s Health.

[3] Jayde V., White K., Blomfield P. (2010). Symptoms and diagnostic delay in ovarian cancer: A summary of the literature. Contemp. Nurse.

[4] McCorkle R., Pasacreta J., Tang S.T. (2003). The Silent Killer: Psychological Issues in Ovarian Cancer. Holist. Nurs. Pract..

[5] Bao P., Bartlett D. (2009). Surgical techniques in visceral resection and peritonectomy procedures. Cancer J..

[6] Calin M.A., Parasca S.V., Savastru D., Manea D. (2014). Hyperspectral Imaging in the Medical Field: Present and Future. Appl. Spectrosc. Rev..

[7] Bhargava A., Sachdeva A., Sharma K., Alsharif M.H., Uthansakul P., Uthansakul M. (2024). Hyperspectral imaging and its applications: A review. Heliyon.

[8] Kho E., Dashtbozorg B., de Boer L.L., Van de Vijver K.K., Sterenborg H.J.C.M., Ruers T.J.M. (2019). Broadband hyperspectral imaging for breast tumor detection using spectral and spatial information. Biomed. Opt. Express.

[9] Lu G., Little J.V., Wang X., Zhang H., Patel M.R., Griffith C.C., El-Deiry M.W., Chen A.Y., Fei B. (2017). Detection of Head and Neck Cancer in Surgical Specimens Using Quantitative Hyperspectral Imaging. Clin. Cancer Res..

[10] Akbari H., Uto K., Kosugi Y., Kojima K., Tanaka N. (2011). Cancer detection using infrared hyperspectral imaging. Cancer Sci..

[11] van Vliet-Pérez S.M., van de Berg N.J., Manni F., Lai M., Rijstenberg L., Hendriks B.H.W., Dankelman J., Ewing-Graham P.C., Nieuwenhuyzen-de Boer G.M., van Beekhuizen H.J. (2022). Hyperspectral Imaging for Tissue Classification after Advanced Stage Ovarian Cancer Surgery—A Pilot Study. Cancers.

[12] Collins T., Maktabi M., Barberio M., Bencteux V., Jansen-Winkeln B., Chalopin C., Marescaux J., Hostettler A., Diana M., Gockel I. (2021). Automatic Recognition of Colon and Esophagogastric Cancer with Machine Learning and Hyperspectral Imaging. Diagnostics.

[13] Dorra N., Yves L., Sylvie T. (2013). Calibration and test of a hyperspectral imaging prototype for intra-operative surgical assistance. Proc. SPIE.

[14] Camps-Valls G., Bruzzone L. (2005). Kernel-based methods for hyperspectral image classification. IEEE Trans. Geosci. Remote Sens..

[15] Guido R., Ferrisi S., Lofaro D., Conforti D. (2024). An Overview on the Advancements of Support Vector Machine Models in Healthcare Applications: A Review. Information.

[16] Taye M.M. (2023). Theoretical Understanding of Convolutional Neural Network: Concepts, Architectures, Applications, Future Directions. Computation.

[17] Krichen M. (2023). Convolutional Neural Networks: A Survey. Computers.

[18] Barberio M., Collins T., Bencteux V., Nkusi R., Felli E., Viola M.G., Marescaux J., Hostettler A., Diana M. (2021). Deep Learning Analysis of In Vivo Hyperspectral Images for Automated Intraoperative Nerve Detection. Diagnostics.

[19] Kozlova E., Chernysh A., Kozlov A., Sergunova V., Sherstyukova E. (2020). Assessment of carboxyhemoglobin content in the blood with high accuracy: Wavelength range optimization for nonlinear curve fitting of optical spectra. Heliyon.

[20] Robbins J.B., Broadwell C., Chow L.C., Parry J.P., Sadowski E.A. (2015). Müllerian duct anomalies: Embryological development, classification, and MRI assessment. J. Magn. Reson. Imaging.

[21] Studier-Fischer A., Seidlitz S., Sellner J., Özdemir B., Wiesenfarth M., Ayala L., Odenthal J., Knödler S., Kowalewski K.F., Haney C.M. (2022). Spectral organ fingerprints for machine learning-based intraoperative tissue classification with hyperspectral imaging in a porcine model. Sci. Rep..

[22] Eggert D., Bengs M., Westermann S., Gessert N., Gerstner A.O.H., Mueller N.A., Bewarder J., Schlaefer A., Betz C., Laffers W. (2022). In vivo detection of head and neck tumors by hyperspectral imaging combined with deep learning methods. J. Biophotonics.

[23] Bannone E., Collins T., Esposito A., Cinelli L., De Pastena M., Pessaux P., Felli E., Andreotti E., Okamoto N., Barberio M. (2024). Surgical optomics: Hyperspectral imaging and deep learning towards precision intraoperative automatic tissue recognition—Results from the EX-MACHYNA trial. Surg. Endosc..

